# Experimental Study on Matched Particle Size and Elastic Modulus of Preformed Particle Gel for Oil Reservoirs

**DOI:** 10.3390/gels8080506

**Published:** 2022-08-14

**Authors:** Kang Zhou, Dejun Wu, Zhibin An

**Affiliations:** 1 College of Energy and Mining Engineering, Shandong University of Science and Technology, Qingdao 266590, China; 2 School of Petroleum Engineering, China University of Petroleum (East China), Qingdao 266580, China

**Keywords:** preformed particle gel, matching relationship, reservoir permeability, sand-pack displacement experiment, microscopic visualization experiment

## Abstract

Suitable elastic modulus and particle size of preformed particle gel are the keys to both diverting water flow and avoiding permanent impairment to reservoirs. Therefore, the paper aims at finding the best matched preformed particle gel for given reservoirs using sand-pack displacement experiments. The results show that the injection pressure of preformed particle gel with excessively small size and elastic modulus is relatively low, indicating poor capacity to increase flow resistance and reduce water channeling. On the other hand, if the particle size and elastic modulus of preformed particle gel are excessively large, the reservoir may be plugged and irreversibly damaged, affecting oil development performance. In fact, the best matched particle size and elastic modulus of preformed particle gel increase with the increase in reservoir permeability. Furthermore, the paper establishes a quantitative logarithmic model between the particle size of preformed particle gel and reservoir permeability. Finally, the established matching relationship is validated via microscopic visualization oil displacement experiments using a glass etching model. The validation experiments indicate that the preformed particle gel (60–80 mesh; 2–4 Pa) selected according to the matching relationship can effectively reduce water channeling and increase sweeping efficiency by as much as 55% compared with water flooding in the glass etching model with an average permeability of 2624 × 10^−3^ μm^2^. Therefore, the established matching relationship can provide an effective guide when selecting the best suitable preformed particle gel for a given reservoir in more future applications.

## 1. Introduction

Most oilfields in China are characterized by high heterogeneity and complex geology, causing oil recovery to be only about 30% after water flooding [1]. In order to enhance oil recovery, chemical methods such as polymer flooding and surfactant flooding are widely used. However, more than 50% of the original oil can still not be produced even if polymer flooding is used [2]. Therefore, more effective fluid-diverting agents attracted research interest around the world in recent years [3]. In fact, preformed particle gels (PPGs) were successfully used to reduce water channeling, increase sweeping efficiency in Shengli Oilfield and obtain great performance even after polymer flooding [4].

PPG enhances oil recovery by temporarily plugging the fluid-channeling paths and forcing the injected water to turn to unswept oil-rich regions [5]. In fact, due to the large fraction of water flow, a large proportion of the injected PPG particles migrate into the channeling regions and may plug the pore throats, especially those with smaller diameters. As a result, subsequent water turns to unswept regions and displaces the remaining oil there. At the same time, the flow resistance and pressure difference gradually increase because of the blockage. Finally, the plugging PPG can deform and pass through the pore throats again when the pressure gradient increases to a certain extent, resulting in another redistribution of the flow paths in the reservoir.

Considering the complex geological conditions of the potential application reservoirs, researchers conducted lots of work to improve the performance of PPGs. Regarding high-temperature and high-salinity conditions, Yu et al. [6] proposed a modified PPG product; Baloochestanzadeh et al. [7] proposed a novel nanocomposite PPG; and Oppong et al. [8] improved the rheological properties and the chemical and thermal stability of PPGs by introducing new functional groups. In terms of the acid environment in supercritical CO2-flooding reservoirs, Zhou et al. [9] proposed an acid-resistant PPG and discussed its conformance-control performance. As for fractured vuggy carbonate reservoirs, Ge et al. [10] evaluated the application of nanocomposite PPGs and discussed the matching relationship between particle size and fracture width. In addition, Malmir et al. [11] focused on the effect of the wettability of reservoir rock on the performance of PPGs using micromodel experiments. Aqcheli et al. [12] obtained a new PPG suspension with improved strength using a new synthetic method and then carried out micromodel oil displacement experiments to evaluate the performance of enhanced oil recovery.

The propagation, retention and restart characteristics are the keys to the success of enhanced oil recovery using PPGs. Regarding propagation, Imqam et al. [13] studied the propagation and plugging mechanisms of PPGs in open void-space conduits with different heterogeneities. Li et al. [14] analyzed the stability, seepage and displacement characteristics of a new branched PPG using sand-pack displacement experiments. Considering PPG retention, Farasat et al. [15] and Saghafi et al. [16] analyzed many influencing factors, including reservoir temperature, displacing-fluid velocity, PPG diameter, flow rate and the porosity of the medium. As for restart, Zhao et al. [17] investigated the restarting pressure gradient of PPGs to deform and pass a pore throat. Matias-Perez et al. [18] studied the pressure-gradient evolution during water flow through a deformable PPG. In the aspect of theoretical and simulation studies, Wang et al. [19] presented a phenomenological model, while Zhou et al. [5,20] developed an efficient LBM-DEM method. More literature works can be found in the latest review papers, including Wu et al. [3], Leng et al. [21] and Esfahlan et al. [22].

Early papers mainly focused on the preparation, propagation and retention characteristics of PPGs from the perspective of theoretical research. However, it is still hard for reservoir engineers to determine the best suitable PPG for a given reservoir from the perspective of practical application. In order to eliminate this problem, the paper aims at finding the best matched PPG size and elastic modulus for different reservoirs using systematic sand-pack displacement experiments. In detail, the paper firstly introduces the experimental materials, apparatus and main procedures for both sand-pack displacement experiments and microscopic visualization oil displacement experiments using a glass etching model. Then, the quantitative matching between PPG and reservoir permeability is established using sand-pack displacement experiments. Finally, the matching relationship is validated using microscopic visualization oil displacement experiments using the glass etching model.

## 2. Experimental Method

### 2.1. Materials

Considering the future application of the matching relationship in practical oilfields, PPG powder samples were taken from Shengli Oilfield. The main agent, initiator, cross-linking agent, additive, reinforcing agent and heat stabilizer were uniformly mixed and cross-linked under ground conditions; thereafter, dry PPG powders were formed via drying and pulverization. Gel strength and particle size are two important parameters to determine PPG performance in increasing sweeping efficiency and enhancing oil recovery. In the experimental study, the range of particle size and elastic modulus were determined according to the industrial PPG product that was available on a large scale and could be used in practical oilfields.

In fact, the elastic moduli of the applied industrial PPGs were 2–4 Pa and 12–16 Pa, respectively. As for each elastic modulus, the particle size distribution of the industrial PPG powders was very wide, and they were classified into 7 groups with different sizes using standard sample sieves for the displacement experiments, including 20–40 mesh, 40–60 mesh, 60–80 mesh, 80–100 mesh, 100–120 mesh, 120–150 mesh and 150–180 mesh. After dry PPG powder swelled in water, their mean diameters were 1664 μm, 1182 μm, 946 μm, 732 μm, 610 μm, 516 μm and 431 μm, respectively. Therefore, the PPG samples used in the experiments formed 14 groups in total.

In the sand-pack displacement experiments, quartz sands were used to prepare the model with a length of 30 cm and a diameter of 2.5 cm. In the microscopic displacement experiments, a square glass etching model with a side length of 2.5 cm was used. The density and viscosity of the experimental oil were 0.89 g/cm^3^ and 50 mPa·s at 25 °C, respectively. The salinity of simulated formation water was 10,000 mg/L.

### 2.2. Sand-Pack-Model Displacement Experiment

**Figure 1:**
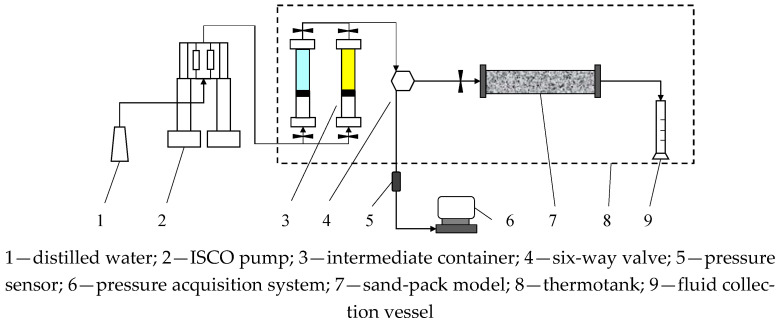
Schematic of sand-pack displacement experimental apparatus.

The matching relationship between PPG and reservoir permeability was studied using sand-pack displacement experiments. Figure 1 shows the experimental apparatus. As can be seen, an ISCO pump, two intermediate containers, a sand-pack model, a pressure and temperature acquisition system, a thermotank and some fluid collection vessels were used. In order to simulate actual reservoir conditions, the thermotank was set to a constant temperature of 70 °C.

The key procedures of the sand-pack displacement experiment are listed as follows: (1) Fill the sand-pack model with a length of 30 cm and a diameter of 2.5 cm with quartz sands. (2) Measure the porosity and permeability of the sand-pack model using the water test method. (3) Inject PPG suspension into the sand-pack model at a constant rate of 0.5 mL/min, which is determined according to the model size and published papers (Li et al., 2018). (4) Collect the injection pressure every 1 min. (5) Stop the injection of PPG suspension when the injection pressure becomes stable or the sand-pack model is plugged. (6) Refill the sand-pack model to obtain a different permeability and repeat the above displacement procedures.

**Figure 2:**
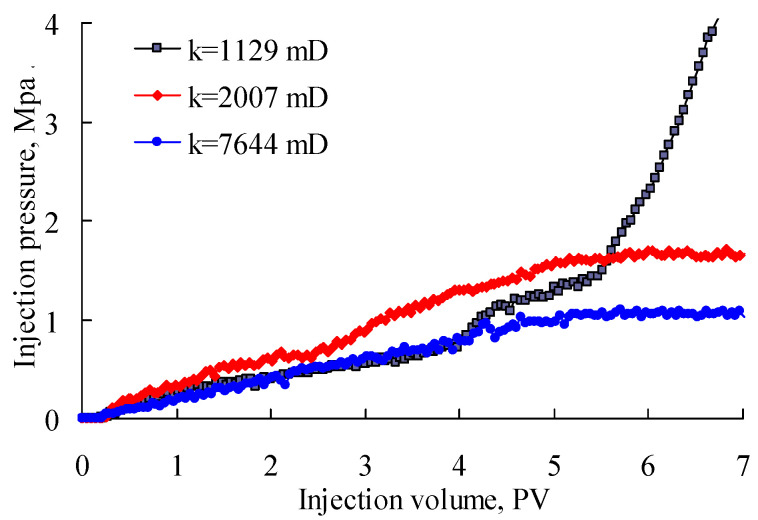
Injection pressure of PPG (80–100 mesh; 2–4 Pa) versus injection volume.

In order to make a good match, PPG samples have to meet two conditions: (1) The PPG can be injected into the reservoir; (2) the PPG can increase the flow resistance of the displacing fluid in water-channeling regions. Therefore, injection pressure can be used to reflect the blockage and remigration ability of PPGs in reservoirs. Figure 2 shows the variation in injection pressure versus injection volume for injecting PPG with the size of 80–100 mesh and the elastic modulus of 2–4 Pa. As can be seen, when the sand-pack permeability was relatively small, such as 1129 × 10^−3^ μm^2^, the injection pressure increased with the injection volume, and the growth rate gradually increased. The phenomenon indicated the sand pack was irreversibly blocked by the injected PPG. Therefore, a PPG with a size of 80–100 mesh and an elastic modulus of 2–4 Pa does not match a reservoir with a permeability of 1129 × 10^−3^ μm^2^. If the sand-pack permeability increased to 2007 × 10^−3^ μm^2^, the injection pressure increased at first and gradually stabilized at 1.6 MPa after 5 PV of PPG was injected. This was mainly because the retention of the PPG in the sand pack became stable and the PPG could continuously deform and migrate in the model. If the sand-pack permeability further increased to a larger value, such as 7644 × 10^−3^ μm^2^, the injection pressure could still stabilize, but it reduced to 1.0 MPa. As for field applications, a good plugging performance is necessary under the condition of injectability. From the experiments, a good matching relationship could be obtained between the 80–100-mesh, 2–4 Pa PPG and the reservoir permeability of 2007 × 10^−3^ μm^2^.

### 2.3. Glass-Etched-Model Oil Displacement Experiment

**Figure 3:**
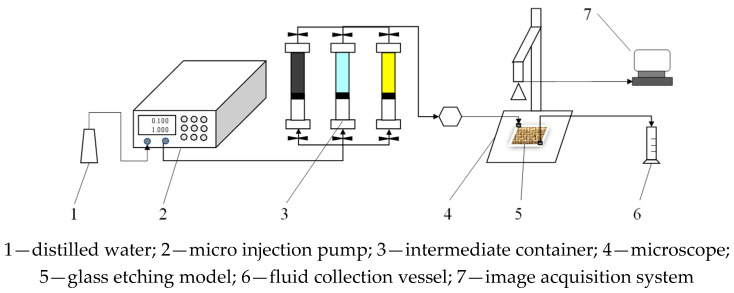
Schematic of microscopic visualization oil displacement experimental apparatus.

In order to validate the established matching relationship between the PPG and reservoir permeability, a glass etching model was used to carry out microscopic visualization oil displacement experiments. Figure 3 shows the schematic of the glass-etched-model oil displacement experimental apparatus. As can be seen, the experiment apparatus mainly contained a micro injection pump, three intermediate containers, a microscope, a glass etching model, fluid collection vessels and an image acquisition system. In the study, the side length of the glass etching model was 2.5 cm. The coordination number and pore-throat ratio were 3 and 1.5, respectively.

The key procedures of the glass-etched-model oil displacement experiment are listed as follows: (1) Clean and vacuumize the glass etching model. (2) Saturate the model with simulated formation water, and measure its porosity and permeability. (3) Saturate the model with crude oil, and age for two hours. (4) Displace the model with simulated formation water for 1 PV at a constant injection rate of 1 µL/min. (5) Displace the model with PPG suspension at a constant injection rate of 1 µL/min until no more oil can be produced. (6) Analyze the displacement images and the obtained quantitative data, and validate the matching relationship.

## 3. Results and Analyses

### 3.1. Matching between PPG and Reservoir Permeability

**Figure 4:**
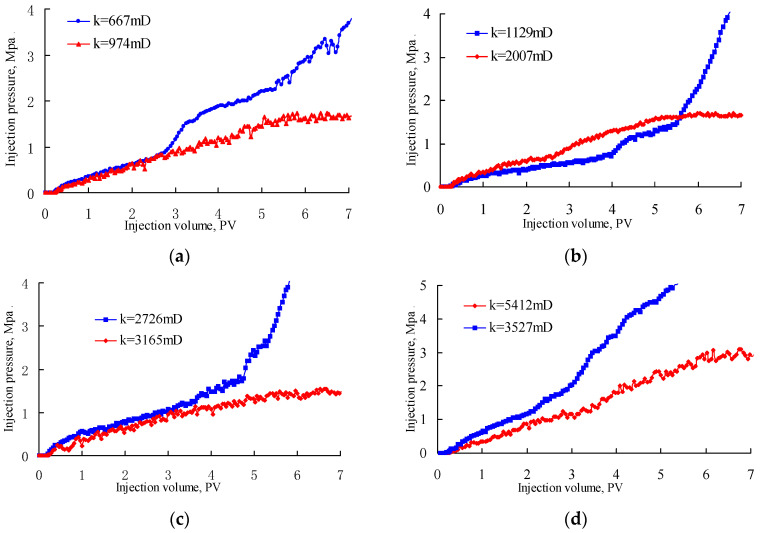
Injection pressure versus injection volume of PPGs (2–4 Pa) with different powder sizes: (**a**) 120–150 mesh, (**b**) 80–100 mesh, (**c**) 40–60 mesh and (**d**) 20–40 mesh.

Figure 4 shows the variation in injection pressure during PPG (2–4 Pa) injection. In these figures, the blue curves indicate irreversible blockage by the injected PPG due to the injection pressure having continuously increased until the experiments were stopped. The red curves indicate that the injection pressure increased at first and gradually stabilized at a certain value, because the retention of the PPG in the sand pack became stable and the PPG could continuously deform and migrate in the model, reflecting good capacity for temporary plugging and deformation ability. As can be seen, the PPG of 120–150 mesh blocked the sand pack with a permeability of 667 × 10^−3^ μm^2^ but performed well when the permeability increased to 974 × 10^−3^ μm^2^. The PPG of 80–100 mesh blocked the sand pack with a permeability of 1129 × 10^−3^ μm^2^ but performed well when the permeability increased to 2007 × 10^−3^ μm^2^. The PPG of 40–60 mesh blocked the sand pack with a permeability of 2726 × 10^−3^ μm^2^ but performed well when the permeability increased to 3165 × 10^−3^ μm^2^. The PPG of 20–40 mesh blocked the sand pack with a permeability of 3527 × 10^−3^ μm^2^ but performed well when the permeability increased to 5412 × 10^−3^ μm^2^. From these experiments, the PPGs (2–4 Pa) with diameters of 120–150 mesh, 80–100 mesh, 40–60 mesh and 20–40 mesh matched well with reservoir permeabilities of 974 × 10^−3^ μm^2^, 2007 × 10^−3^ μm^2^, 3165 × 10^−3^ μm^2^ and 5412 × 10^−3^ μm^2^, respectively.

**Figure 5:**
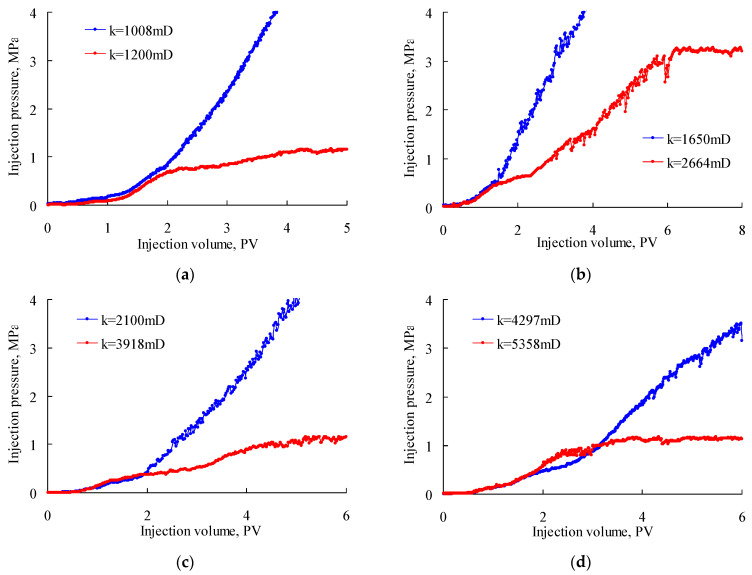
Injection pressure versus injection volume of PPGs (12–16 Pa) with different powder sizes: (**a**) 120–150 mesh, (**b**) 100–120 mesh, (**c**) 80–100 mesh and (**d**) 60–80 mesh.

Figure 5 shows the variation in injection pressure during PPG (12–16 Pa) injection. Similar to Figure 4, the blue curves indicate irreversible blockage by the injected PPG, while the red curves reflect good capacity for temporary plugging and deformation ability. As can be seen, the PPG of 120–150 mesh blocked the sand pack with a permeability of 1008 × 10^−3^ μm^2^ but performed well when the permeability increased to 1200 × 10^−3^ μm^2^. The PPG of 100–120 mesh blocked the sand pack with a permeability of 1650 × 10^−3^ μm^2^ but performed well when the permeability increased to 2664 × 10^−3^ μm^2^. The PPG of 80–100 mesh blocked the sand pack with a permeability of 2100 × 10^−3^ μm^2^ but performed well when the permeability increased to 3918 × 10^−3^ μm^2^. The PPG of 60–80 mesh blocked the sand pack with a permeability of 4297 × 10^−3^ μm^2^ but performed well when the permeability increased to 5358 × 10^−3^ μm^2^. From these experiments, the PPGs (12–16 Pa) with particle sizes of 120–150 mesh, 100–120 mesh, 80–100 mesh and 60–80 mesh matched well with reservoir permeabilities of 1200 × 10^−3^ μm^2^, 2664 × 10^−3^ μm^2^, 3918 × 10^−3^ μm^2^ and 5358 × 10^−3^ μm^2^, respectively.

**Figure 6:**
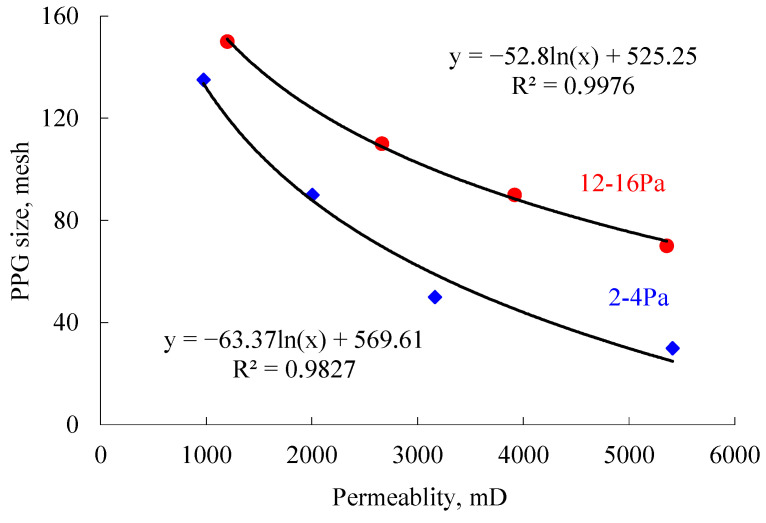
Matching relationships among PPG powder size, elastic modulus and reservoir permeability.

Based on the experiments detailed above, the matching relationship could be obtained, as shown in Figure 6. When the elastic modulus of a PPG is kept unchanged, the matching mesh of the PPG decreases as the permeability increases. In other words, the larger the permeability is, the larger the matching PPG size needed is. This is mainly because the pore-throat diameter is larger in reservoirs with larger permeability. Under conditions of similar deformation, the allowed PPG size is larger. When the PPG mesh is kept unchanged, the matching elastic modulus of the PPG increases with the increase in reservoir permeability. Using a regression analysis, the matching PPG mesh and reservoir permeability exhibit a logarithmic relation, and the relation coefficient reaches more than 98%. From the figure, PPGs with larger particle sizes and elastic moduli should be selected in order to improve the development performance under the condition that they can be injected.

### 3.2. Oil Displacement Performance of Matching PPG

In the microscopic visualization oil displacement experiments, a glass etching model with a coordination number of 3 and a pore-throat diameter ratio of 1.5 was used. The permeability of the glass etching model was 2624 × 10^−3^ μm^2^, measured using the water test method. According to the established matching relationship between PPG and reservoir permeability, the mean size of the best matched PPG had to be 71 mesh (2–4 Pa) or 110 mesh (12–16 Pa). Considering the 14 groups of PPG samples classified in the sand-pack experiment, the paper selected the PPG of 2–4 Pa and 60–80 mesh as the best matched fluid-diverting agent. For the sake of contrast, the PPG sample of 2–4 Pa and 120–150 mesh was also used to carry out the oil displacement experiments.

**Figure 7:**
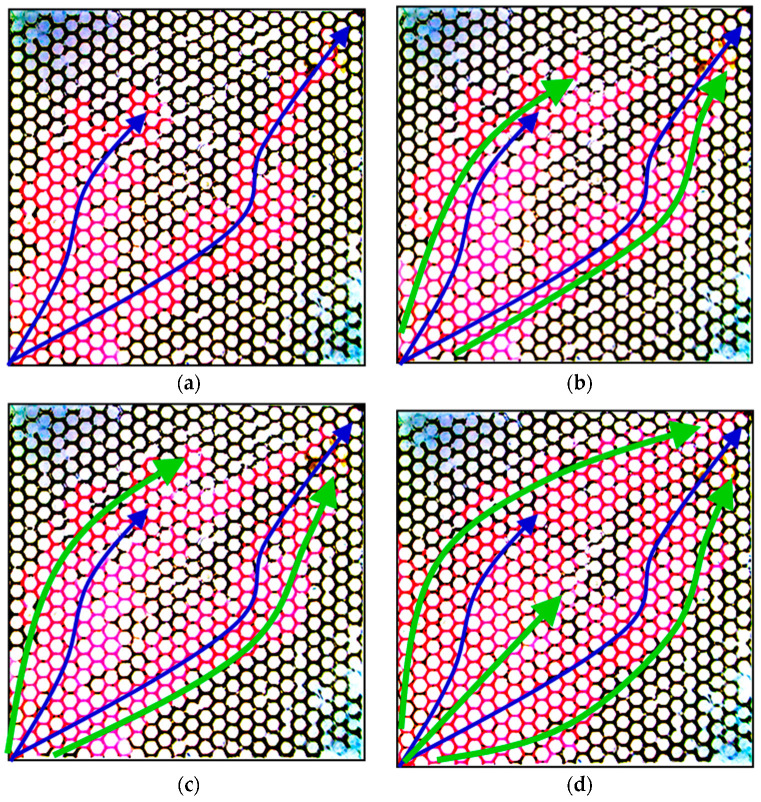
Micrographs of oil displacement experiment using PPG of 2–4 Pa, 120–150 mesh: (**a**) end of water flooding, (**b**) 0.25 PV of PPG flooding, (**c**) 0.5 PV of PPG flooding and (**d**) end of PPG flooding.

Figure 7 shows the micrographs of the oil displacement experiment using the PPG of 2–4 Pa and 120–150 mesh. The displacing fluid was injected from the left bottom corner, and the liquid was produced from the top right corner. In these figures, the blue arrows represent the main streamlines during the water-flooding stage, and the green arrows represent the main streamlines during the PPG-flooding stage. As can be seen, the injected water mainly flowed along the diagonal streamline and formed an obvious fingering phenomenon. At the end of water flooding, the sweeping efficiency was only 33%, and there was still a lot of oil remaining in the model, as shown in Figure 7a. After injecting the PPG (2–4 Pa, 120–150 mesh) suspension, the sweeping efficiency was slightly increased due to the fluid-diverting performance of the PPG. At the end of PPG flooding, the sweeping efficiency reached 59%, which was 26% higher than that of water flooding. However, as can be seen from Figure 7d, several large parts of the glass etching model were still unswept, and much oil could still not be produced. This is mainly because the PPG sample of 120–150 mesh was much smaller than that needed to block the water-channeling region in the model with a permeability of 2624 × 10^−3^ μm^2^; thus, it could not force the injected suspension to flow towards the oil-rich areas.

**Figure 8:**
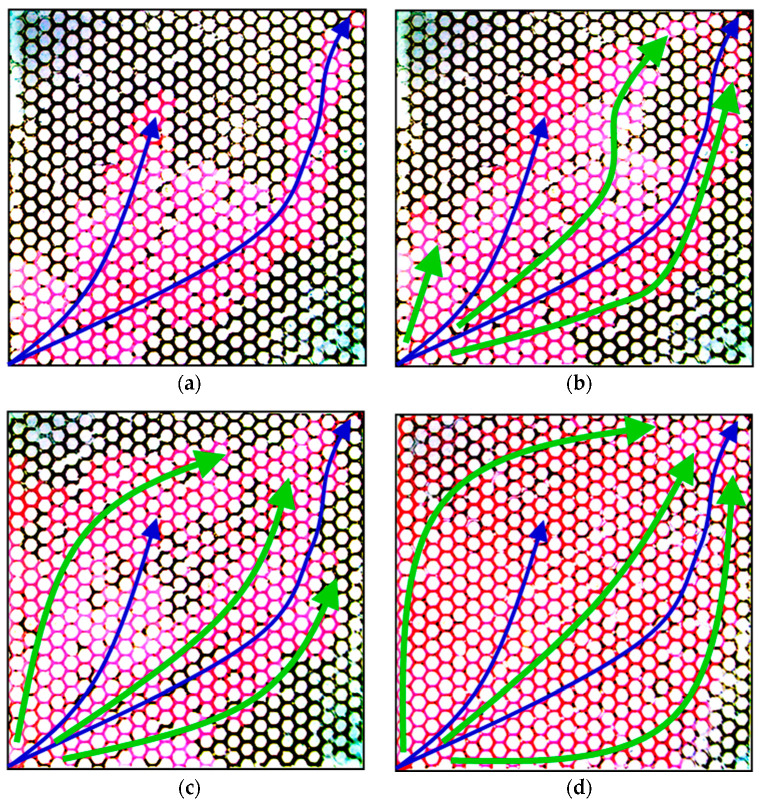
Micrographs of oil displacement experiment using PPG of 2–4 Pa, 60–80 mesh: (**a**) end of water flooding, (**b**) 0.25 PV of PPG flooding, (**c**) 0.5 PV of PPG flooding and (**d**) end of PPG flooding.

Figure 8 shows the micrographs of the oil displacement experiment using the PPG of 2–4 Pa and 60–80 mesh. In these figures, the blue arrows represent the main streamlines during the water-flooding stage, and the green arrows represent the main streamlines during the PPG-flooding stage. Similar to Figure 7, there was severe water channeling during water flooding, and the sweeping efficiency was only 36% at the end of the 1 PV injection of simulated water. However, with the injection of the PPG (2–4 Pa, 60–80 mesh) suspension, the sweeping efficiency largely increased and reached 61% after injecting 0.5 PV of PPG suspension. This was mainly because the PPG of 60–80 mesh was much larger than the PPG of 120–150 mesh, causing larger flow resistance and forcing the displacing fluid to change to the unswept region around the water-channeling diagonal streamlines. At the end of PPG flooding, most of the glass etching model had been swept, and the sweeping efficiency reached 91%, which was 55% higher than that of water flooding. Comparing Figure 7 and Figure 8, the sweeping efficiency increased using the PPG of 60–80 mesh was 29% higher than that increased using the PPG of 120–150 mesh.

By comparing the oil displacement results as shown in Figure 7 and Figure 8, the PPG elastic modulus and particle size determined according to the matching relationship could effectively block the water-channeling regions and increase the sweeping efficiency. On the other hand, the matched PPG size was not too large and thus avoided the permanent plugging of the glass etching model, which may have caused worse development performance. So, the selected PPG met the two conditions for matching with reservoir permeability as mentioned in Section 2.2. Therefore, the established matching relationship is dependable when it is used to determine suitable PPG size and elastic modulus according to reservoir permeability.

## 4. Conclusions

(1) For a given reservoir with a certain permeability, a PPG with excessively small size and elastic modulus has poor capacity to increase flow resistance and reduce water channeling. A PPG with excessively large size and elastic modulus may cause permanent plugging and irreversible impairment to the reservoirs. The larger the reservoir permeability is, the larger the best matched PPG size and elastic modulus needed are.
(2) The best matched PPG size and reservoir permeability have a positive logarithmic relationship, and the correlation coefficient reaches over 98%. Microscopic visualization experiments using a glass etching model prove that a PPG of 60–80 mesh and 2–4 Pa selected according to the logarithmic relationship can increase the sweeping efficiency by as much as 55% compared with water flooding for a reservoir with a permeability of 2624 × 10^−3^ μm^2^.

## Data Availability

Data available on request from the authors.
